# The effect of multimodal care based on Peplau’s interpersonal relationship theory on postoperative recovery in lung cancer surgery: a retrospective analysis

**DOI:** 10.1186/s12890-024-02874-5

**Published:** 2024-01-27

**Authors:** Xue-e Su, Shan-hu Wu, He-fan He, Cui-liu Lin, Shu Lin, Pei-qing Weng

**Affiliations:** 1 Centre of Neurological and Metabolic Research, The Second Affiliated Hospital of Fujian Medical University, No.34 North Zhongshan Road, Quanzhou, Fujian Province 362000 China; 2 Group of Neuroendocrinology, Garvan Institute of Medical Research, 384 Victoria St, Sydney, Australia; 3 Department of Anesthesia, The Second Affiliated Hospital of Fujian Medical University, No.34 North Zhongshan Road, Quanzhou, Fujian Province 362000 China

**Keywords:** Peplau’s interpersonal relationship theory, Anesthetic nursing model, Lung cancer surgery

## Abstract

**Background:**

Lung cancer remains a major global health concern due to its high incidence and mortality rates. With advancements in medical treatments, an increasing number of early-stage lung cancer cases are being detected, making surgical treatment the primary option for such cases. However, this presents challenges to the physical and mental recovery of patients. Peplau known as the “mother of psychiatric associations” has formulated a theory of interpersonal relationships in nursing. Through effective communication between nurses and patients over four periods, she has established a good therapeutic nurse-patient relationship. Therefore, this study aimed to explore the effect of perioperative multimodal nursing based on Peplau’s interpersonal relationship theory on the rehabilitation of patients with surgical lung cancer.

**Methods:**

We retrospectively analyzed 106 patients with non-small cell lung cancer who underwent thoracoscopic lobectomy at our department between June 2021 and April 2022. Patients were categorized into two groups according to the different nursing intervention techniques. The Peplau’s group comprised 53 patients who received targeted nursing interventions, and the control group comprised 53 patients who received conventional nursing care. We observed the patients’ illness uncertainty, quality of life, and clinical symptoms in both groups.

**Results:**

Patients in the Peplau’s group had significantly lower illness uncertainty scores and a significantly higher quality of recovery than those in the control group. However, there were no significant differences in length of post-anesthesia care unit stay, complication rates, and visual analog scores between both groups.

**Conclusion:**

The multimodal perioperative nursing based on Peplau’s interpersonal relationship theory not only reduces the illness uncertainty of patients with lung cancer surgery and improves their QoR but also expands the application of this theory in clinical practice, guiding perioperative nursing of patients with lung cancer.

**Implications:**

These findings provide practical information for standardized care in a hectic anesthetic care setting.

**Impact:**

The assessed anesthesia nursing model helps reduce uncertainty and promote early recovery in patients with cancer at various stages of their disease, which expands the scope of therapeutic practice and existing theories. It also serves as a guide for care in the anesthesia recovery room.

**Reporting method:**

We adhered to the relevant Equator guidelines and the checklist of items in the case–control study report.

**Patient or public contribution:**

Patients cooperated with medical staff to complete relevant scales.

## Global contribution

•Demand for nursing care from patients with NSCLC at various stages of the disease varies and changes over time.
•The anesthesia nursing model based on Peplau’s interpersonal relationship theory helps to reduce uncertainty and promote early recovery in patients with cancer.
•This model has some viability and efficacy, which expands the scope of therapeutic practice and existing theories. It also serves as a guide for care in the anesthesia recovery room.

## Background

According to the latest medical information, lung cancer continues to exhibit high incidence and mortality rates worldwide, posing a serious threat to human health [1]. However, with the rapid advancements in modern medical treatments, an increasing number of lung cancers are being detected at an early stage. Surgical treatment is considered the primary treatment option for early-stage non-small cell lung cancer (NSCLC) [2]. Although surgical intervention can be lifesaving, patients also face enormous physiological and psychological pressure during the perioperative period, necessitating optimal nursing care throughout the treatment process to improve patient outcomes.

While pertinent literature has contributed to the advancement of anesthesia care during the rapid development of modern anesthesia medicine in China, several anesthesia nursing procedures remained in the exploratory stage when rules and regulations were being established. Therefore, medical professionals should provide appropriate nursing care to assist patients throughout the treatment process and improve their prognosis.

Numerous studies have demonstrated that effective health intervention can lead to positive mood enhancement, reduced complication, and improved quality of life in patients with NSCLC [3–6]. However, compared to developed countries, anesthesia nursing started relatively late in China, and related anesthesia nursing service models were immature. Currently, the implementation of health nursing intervention measures still lacks anesthesia nurse-led care, particularly for patients undergoing surgical treatment. Thus, exploring related nursing models is essential for the development of continuous nursing. Additionally, traditional health education in China is disease-focused and frequently disregards the unique requirements of patients with varying stages of disease. This frequently results in prejudice while giving and receiving medical information, which partially lessens the efficiency of health education. The timing and content of pertinent health education materials are inconsistent with the current nursing needs of patients. Furthermore, health interventions for perioperative patients in China are relatively limited in scope, overly simplistic in form, and lack dynamic psychological assessment and intervention.

Hildegard E. Peplau, a renowned nursing theorist, proposed Peplau’s theory of interpersonal relationships [7]. It emphasizes the establishment of a harmonious, mutually understanding, and respectful nurse-patient relationship to gain a broader understanding of patient problems and proposes practical approaches, through the following four dynamic stages of nurse-patient communication: recognition, determination, progress, and resolution [8]. Although this theory has been widely applied to patients with chronic diseases and nursing education, its application in perioperative patients, particularly in anesthesia nurse-led care for lung surgery patients, remains scarce.

Therefore, based on the role of Peplau’s interpersonal relationship theory, the physiological and psychological rehabilitation needs of patients with surgical lung cancer during the perioperative period, and the background of anesthesia nursing work, we proposed the following scientific hypothesis: multimodal perioperative anesthesia nursing based on Peplau’s interpersonal relationship theory can promote the physiological and psychological rehabilitation of patients with lung cancer. To verify the above hypothesis, we conducted a retrospective study to observe the impact of multi-modal anesthesia nursing under the guidance of this theory on illness uncertainty, quality of recovery (QoR), and clinical symptoms in patients with lung cancer. This study holds great significance for the application of this theory in the field of anesthesia nursing and the rehabilitation of patients with lung cancer.

## Methods

In this study, we adopted Peplau’s interpersonal theory as a framework to focus on the care needs of perioperative patients. We intended to understand the effectiveness of this care model on the health outcomes of these patients. Additionally, we aimed to improve the clinical outcomes of patients by reducing their illness uncertainty and enhancing early recovery while providing a theoretical and practical basis for the application of Peplau’s interpersonal theory in perioperative anesthesia care.

### Design

This study used a retrospective observational design involving a retrospective analysis of patients who underwent radical lung cancer resection.

### Study setting and sampling

**Fig. 1:**
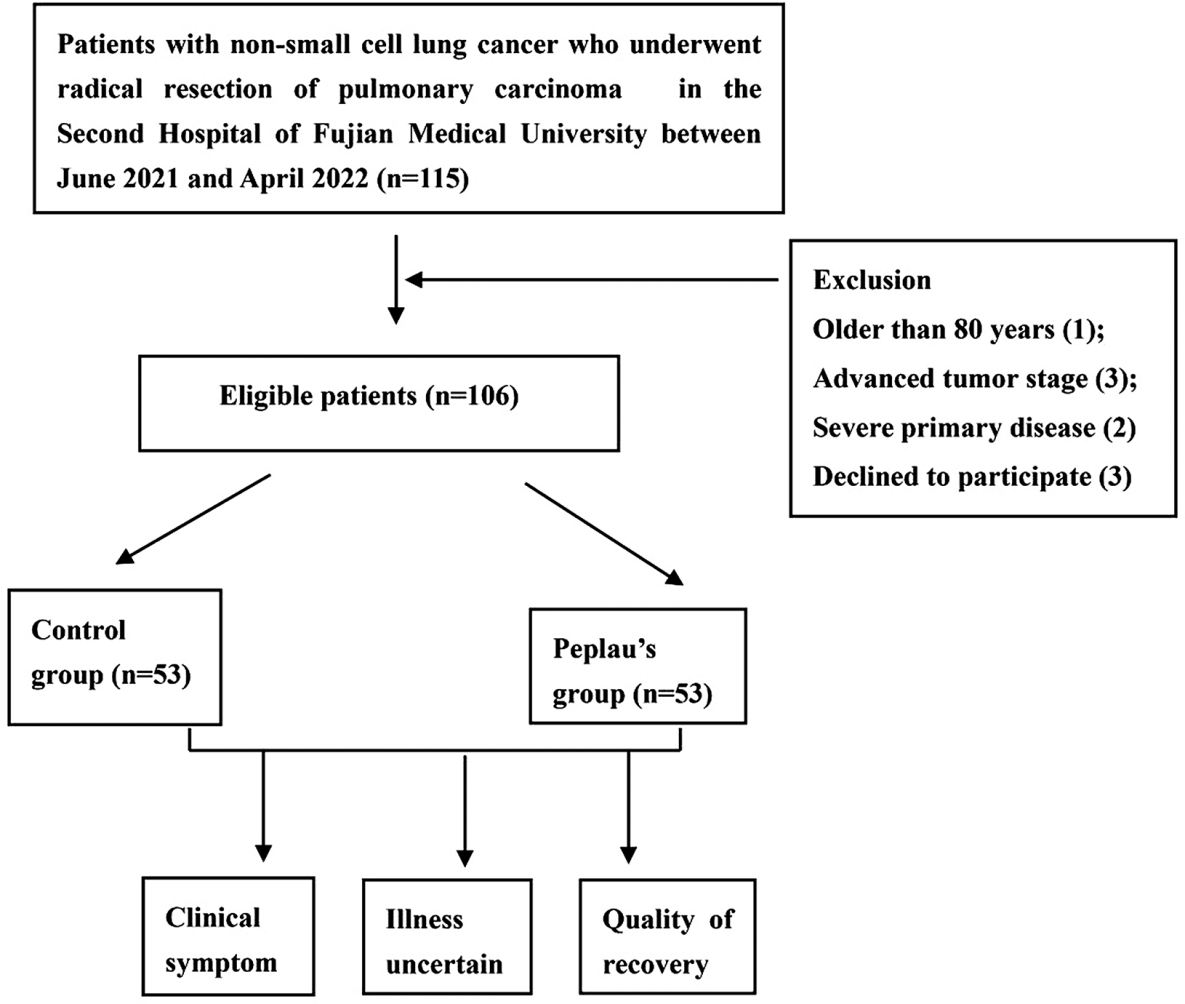
Flow chart of the included patients

One hundred and six patients who underwent radical lung cancer resection at the Second Hospital of Fujian Medical University between June 1, 2021, and April 30, 2022, were retrospectively selected (Fig. 1). These patients were categorized into two groups due to the implementation nodes of multimodal perioperative nursing based on Peplau’s interpersonal relationship theory. The Peplau’s group (PG) comprised 53 patients who received multimodal care based on Peplau’s interpersonal relationship theory, while the control group (CG) comprised 53 individuals who received conventional nursing interventions. Specific intervention measures are detailed in Table 1.

**Table 1:** Comparison of intervention measures between the two groups

Group	Intervention time	Intervention measures	Interveners	Intervention characteristics
CG	•Before surgery	Short preoperative anesthesia conversation to understand the patient’s physical and mental condition	anesthesia nurse	Different anesthesia nurses provide care according to the needs of patients at different stages
	•During surgery	good preparation for anesthesia and regular intraoperative observation of changes in the patient’s vital signs	anesthesia nurse	
	•After surgery	continuous monitoring of the patient’s vital signs in the recovery area after surgery, and follow-up visits to the ward to address the patient’s pain	anesthesia nurse	
	•Recognition:	Comprehensive physical assessment to help patients understand the risks of anesthesia; Provide psychological support for patients; Share successful cases of surgical anesthesia to enhance trust; Provide indications for deep breathing, coughing, etc.	The same anesthesia nurse	**The same nurse** (even if sometimes unable to do so, feedback the patient’s basic situation to the next nurse) provides **personalized, detail and procedural care** at different stages of the patient, **dynamically** meeting the patient’s nursing needs
	•Determination	Meet with the patient again to alleviate their anxiety in an unfamiliar environment, and prepare for anesthesia, including preparing medication and inserting intravenous or other catheters	The same anesthesia nurse	
PG	•Progression	Based on the previous two interactions with the patient, we have gained an understanding of their basic situation and provided more personalized anesthesia recovery room care, including vital sign monitoring, psychological needs satisfaction, and evaluation of recovery room standards, etc.	The same anesthesia nurse	
	•Resolution	On the basis of multiple communications with patients in the early stage, nursing care, and building trust, AIDET communication was adopted for postoperative follow-up to address patient pain and understand their needs	The same anesthesia nurse	

CG: Control group; PG: Peplau’s group; AIDET: Acknowledge; introduce; duration; explicit; thank you

### Inclusion criteria

To be included in this study, patients had to meet the following criteria: confirmed diagnosis of NSCLC through pathological analysis, stages I–II (T_1 − 3_N_0_M_0_ and T_1 − 2_N_1_M_0_); age between 18 and 80 years; stable vital signs and complete baseline data; thoracoscopic lobectomy without prior treatment, chemotherapy, or other adjuvant medication; and normal cognitive function and verbal communication to understand and complete the assessment scale.

### Exclusion criteria

The following patients were excluded from the study: those merged with other cancers; major systemic diseases affecting the heart, brain, kidney, or other organs; psychiatric and cognitive disorders; concurrent serious infectious diseases; and those who withdrew from the study for various reasons or were unable to cooperate.

### Nursing care methods

In the CG, patients received the following routine nursing care: (1) active pre-operative conversation with the patient, explaining anesthetic instructions, and encouraging active participation in the procedure; (2) good preparation for anesthesia and regular intraoperative observation of changes in the patient’s vital signs; (3) administration of multimodal complex analgesia and continuous monitoring of the patient’s vital signs in the recovery area after anesthesia; and (4) follow-up visits to the ward to address the patient’s pain.

In the PG, patients received specialized care in four phases as follows:

1. Recognition: preoperative ward assessment

A day prior to the surgery, a nurse anesthetist visited the ward to conduct a pre-anesthetic examination. This bedside communication included a comprehensive assessment of the patient’s respiratory and cardiovascular system, enabling both nurses and patients to fully understand the risks associated with anesthesia [9]. Psychological care was provided to the patients and their families to further explain the general process of anesthesia and address matters related to cooperation. Successful cases were shared to alleviate psychological burdens, minimize uncertainty about the disease, build trust between patients and nurses, increase patient confidence, and promote positive cooperation. Additionally, clear instructions on coughing and deep breathing were provided to help patients recover quickly, which benefits both recovery from anesthesia and the disease. This period is the beginning stage of therapeutic nurse-patient relationships based on the Peplau theory, and nurses should focus on building trust relationships with patients.

(2) Determination: evaluation of the anesthesia preparation room

The anesthetic preparation room serves as the setting where the anesthetist and the nurse prepare for anesthesia. Under the guidance of the anesthetist and considering the patient’s risk factors, the anesthetic nurses prepared drugs, performed venous and arterial punctures, and monitored vital signs. Nurses ensured that patients received reassurance and addressed their main concerns to alleviate any anxiety that may arise in an unfamiliar environment, which particularly emphasized that the nurse who conducted preoperative visits to the patient the day before reappeared to answer patient’s questions and alleviate their anxiety in unfamiliar environments.

(3) Progression: nursing care in the postoperative anesthesia monitoring room

The recovery room, functioning as a central monitoring unit, plays a crucial role in perioperative patients care [10]. Despite continuous monitoring, there is a high potential for issues in patients during their stay in this area, mainly due to prior anesthesia and surgery. Therefore, nurses in the recovery room adjust the room temperature and provide thermal blankets and heaters to avoid complications, shivering, and awakening based on each patient’s preoperative vital signs, including temperature, pulse, respiration, blood pressure, and other factors such as the preoperative situation, particular anesthetic situation, and the current status of the operation. Additionally, as the primary caregivers during the immediate postoperative period, nurses pay close attention to the patient’s pain management and care of drainage tubes. Once the patient awakens and meets the discharge criteria based on modified Aldrete or Steward scoring(the scoring criteria used to evaluate the recovery status of patients undergoing general anesthesia surgery, including indicators such as consciousness, breathing, and circulation), the nurse accompanies the patient back to the ward and provides detailed explanations to both the patient and their family members, ensuring the continuity of nursing care. During this period, it is particularly emphasized that nurses carry out nursing based on the trust established during preoperative visits and preparation with patients, meet the needs of patients during anesthesia recovery, and further establish a sense of trust.

(4) Resolution: postoperative ward visit

Postoperative visits are essential for implementing a multidisciplinary approach to comprehensive care. Although the reduction in the incidence of postoperative pain is significantly lower using multimodal analgesia, it remains a major concern for patients. Therefore, anesthetic nurses prioritize pain management during follow-up [11], including the interpretation and management of common complications, such as nausea and vomiting. Patients gradually recovered from surgery starting from the first day until their discharge from the hospital, allowing them to interact regularly with nurses. In the late 1990s the American Baptist Memorial Hospital pioneered the use of AIDET (A: acknowledge; I: introduce; D: duration; E: explicit; T: thank you) as a form of communication, which was later adopted in China for anesthetic pain management. This approach not only effectively controls pain but also enhances mutual trust between nurses and patients while increasing patients’ and doctors’ engagement in pain management [12]. Our anesthetic nurses used the AIDET standardized communication model for postoperative follow-up. They addressed individual patients’ concerns and focused on preventing complications and facilitating rehabilitation after discharge. During this period, the therapeutic nurse-patient relationship between nurses and patients tends to come to an end, and special emphasis should be placed on gratitude for patient cooperation, encouraging patients to actively recover and gradually return to normal life.

### Outcome measures

#### Illness uncertainty scores

Illness uncertainty refers to a person’s inability to comprehend events related to their illness and has been identified as a prevalent source of psychological stress in chronic diseases. According to illness uncertainty theory, patients may experience uncertainty when they are unable to categorize the significance of events linked to their condition, such as symptoms and treatment outcomes [13]. This outcome was measured using the Mishel Uncertainty in Illness Scale (MUIS) [14]. The MUIS comprises of 33 items categorized into four dimensions as follows: uncertainty regarding sickness status (13 items), complexity (7 items), lack of information (7 items), and unpredictable course and outcome of illness (5 items). Each item was scored on a 5-point Likert scale, with 1 indicating strong disagreement and 5 representing strong agreement. Higher scores reflected a higher level of disease uncertainty. The overall scores range from 32 to 160, categorized into low (32–74.7), moderate (74.8–117.4), and high (117.5–160) [15]. The scores were collected indicators from patients after surgery and the day before discharge.

#### Quality of recovery

Quality of recovery (QoR) is a fundamental concept in perioperative patient care. The QoR-15 is a questionnaire that evaluates QoR in five areas: pain, physical independence and comfort, psychological support, and emotional state. It has been validated for assessing inpatient and ambulatory anesthesia during the intermediate recovery period [16, 17]. The QoR-15 score ranges from 0 to 150 representing poor to excellent recovery. The scores are categorized as excellent, medium, moderate, and poor recovery [18]. According to patients, clear communication, active participation in healthcare decisions, and compassion from healthcare professionals are considered important elements of the quality of their recovery. The scores were collected indicators from patients after surgery and the day before discharge.

#### Clinical outcomes

We recorded the length of post-anesthesia care unit (PACU) stay, length of hospital stay, visual analog scale (VAS) scores, and occurrence of PACU and postoperative complications. Early hypoxemia is the most frequent complication in the radical resection of lung cancer. Previous studies have shown that hypoxemia is more severe on post-operative days 1 and 3 [19, 20]. Emergence agitation, characterized by inappropriate behavior during the awakening period from general anesthesia, is another common complication of anesthesia, reported in 11–51% of cases [21, 22]. The VAS scores were collected from patients at 24, 48 and 72 h postoperatively.

### Data collection and analysis

The data were analyzed using IBM SPSS Statistics for Windows, version 20.0 (IBM Corp., Armonk, N.Y., USA) to ensure accuracy. The Shapiro–Wilk test was applied to establish normal distribution. Descriptive statistics were presented using the median for non-normally distributed data, while the mean (X̄) ± standard deviation (SD) was used for variables with a normal distribution. Categorical variables were presented as numbers and percentages. The significance of the difference between the two means for the continuous variables was assessed in the PG and CG using the Student’s t -test, a parametric test for normally distributed values, and the Mann–Whitney U test for non-normally distributed data. The analysis of categorical variables employed Pearson’s chi-square test. Statistical significance was set at *P* < 0.05.

### Ethical considerations

This study was conducted in compliance with the principles of the Declaration of Helsinki. Owing to the retrospective nature of this study, the need for informed consent was waived by the Medical Ethics Committee of the Second Affiliated Hospital of Fujian Medical University, and the study design was approved by the Medical Ethics Committee of the Second Affiliated Hospital of Fujian Medical University on January 7,2023 (No. 2,023,125).

## Results

### Patients characteristics

The PG comprised patients aged 27–75 years, with an average of (53.62 ± 11.43) years, whereas the CG included those aged 28–80 years, with a mean age of (56.24 ± 12.28) years. The differences in mean age, body mass index, sex, Tumor Node Metastasis (TNM) stage, education, smoking history, family history, and alcohol consumption between the PG and CG were not significant (all *P* > 0.05; Table 2).

**Table 2:** Patient characteristics and clinical characteristics

Patient data	PG	CG
Male [n (%)]	18 (33.96%)	26(49.06%)
Adenocarcinoma [n (%)]	50 (94.34%)	51(96.22%)
Squamous carcinoma [n (%)]	3 (9.43%)	2(3.77%)
History of hypertension [n (%)]	15 (28.30%)	13(24.53%)
Diabetes mellitus [n (%)]	7 (13.21%)	9(16.98%)
History of coronary heart disease [n (%)]	3 (5.66%)	2(3.77%)
History of surgery [n (%)]	9 (16.98%)	11(20.75%)
TNM stage (I) [n (%)]	30 (56.60%)	27(50.94%)
TNM stage (II) [n (%)]	23 (43.39%)	26(49.05%)
Age (year,$\bar x$±s) [n (%)]	53.62 ± 11.43	56.25 ± 12.28

PG: Peplau’s group; CG: control group; TNM: Tumor Node Metastasis

### Comparison of illness uncertainty scores after intervention in both the groups

Illness uncertainty was observed in both the groups. The results indicated that the post-operative scores were significantly lower in both groups, and disease uncertainty, complexity, lack of information, and unpredictability scores were significantly lower in the PG than those in the CG (*P* < 0.05; Table 3).

**Table 3:** Post-intervention scores of illness uncertainty

	PG(*n* = 53)	CG(*n* = 53)	t/Z	P
Total scores	91.87 ± 4.47	106.34 ± 4.86	15.95	< 0.01
Uncertainty	34.60 ± 2.78	48.17 ± 2.80	25.02	< 0.01
Complexity	24.49(23.00–26.00)	18.87(17.00–20.50)	8.42	< 0.01
Lack of information	19.02(18.00–20.00)	25.02(24.00–26.00)	-8.42	< 0.01
Unpredictable	13.75(13.00–14.00)	14.28(13.00–15.00)	-2.07	0.03

PG: Peplau’s group; CG: control group

### Comparison of the quality-of-life levels between both groups after the intervention

The rehabilitation levels of both groups were evaluated after the intervention. The results indicated that the QoR score was 97 (94–100) and 93 (92–97) in the PG and CG, respectively. The comparison showed that the QoR in the PG was significantly higher than that in the CG (*P* < 0.05; Table 4).

**Table 4:** Post-intervention scores of quality of life

	PG(*n* = 53)	CG(*n* = 53)	Z	P
Total scores	97(94–100)	93(92–97)	6.07	< 0.01
Physical comfort	34(32–34)	33(31–34)	1.92	0.06
Physical independence	11(12–14)	12(12–13)	6.45	< 0.01
psychological support	16(15–16)	18(16–18.5)	7.58	< 0.01
Emotional stat	25(25–26)	24(23–26)	-2.91	0.004
Pain	11(10–12)	10(8–11)	-1.529	0.13

PG: Peplau’s group; CG: control group

### Clinical symptoms of patients in both groups after the intervention

**Fig. 2:**
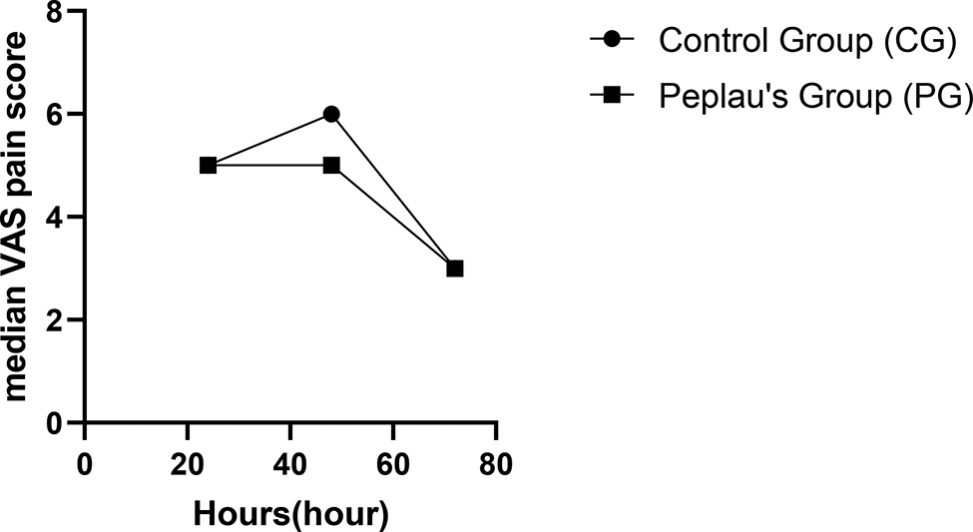
Patient’s postoperative pain score

Clinical symptoms were observed in both the groups. No significant differences were observed between the two groups in terms of the length of PACU stay, hospital stay, complication rate, and VAS scores (Table 5; Fig. 2).

**Table 5:** Clinical symptom during the study period

	PG(*n* = 53)	CG(*n* = 53)	Z/χ^2^	P
Length of PACU stay, min	50(45–54)	51(48–55)	-1.670	0.095
The length of hospitalization, min	9(6–11)	9(7–11)	-0.321	0.748
Occurrence of PACU complication, n (%)	3(5.7%)	4(7.5%)	0.217	0.641
Occurrence of postoperative complication, n (%)	11(20.8%)	10(18.9%)	0.059	0.807

PG: Peplau’s group; CG: control group; PACU: post-anesthesia care unit

## Discussion

This retrospective study revealed that compared to patiens receiving routine care, those who received multimodal perioperative care based on Peplau’s interpersonal relationship theory exhibited significantly improved illness uncertainty and QoR in the subjects. However, the multimodal perioperative care based on Peplau’s interpersonal relationship theory was not statistically significance for the patient’s length of PACU stay, length of hospitalization, postoperative VAS score, PACU complications, and incidence of postoperative complications.

During the second half of the 19th century, anesthesia care flourished in the United States owing to a lack of qualified anesthesiologists [23]. China started anesthesia nursing relatively late and has recently released a detailed strategy to improve the standards of anesthetic care and ensure patient safety and comfort. Consequently, nursing anesthetists are receiving increasing attention, emphasizing the importance of creating and implementing targeted training and clinical practice [24]. Therefore, it is crucial to establish a standardized approach to anesthesia care to inform the anesthesia care profession and improve the consistency, quality, and efficiency of care.

An individual’s inability to explain disease-related events, known as illness uncertainty, has been identified as a common source of psychological stress in chronic diseases. Patients with lung cancer often face challenges related to higher levels of physical symptom distress, mental health and daily living challenges, and burdensome symptoms compared to patients with other types of cancer. This is due to the high morbidity and mortality associated with lung cancer during and after treatment [25, 26]. All the respondents experienced frustration upon learning about their cancer diagnoses. It heralds the transition from a healthy person to a patient and to a time of perpetual change and difficulty. In addition, the extensive pre-operative testing that patients undergo upon hospital admission leads to increased levels of anxiety and stress. Most studies (65%) found that interventions targeting disease uncertainty management had favorable effects on uncertainty outcomes. Multicomponent therapies, which integrate informational and emotional support, appear to be the most successful approach to managing illness uncertainty in patients with cancer and their family caregivers [27, 28]. Therefore, a new and effective intervention model for peripheral surgical anesthesia is urgently needed.

Peplau’s theory of interpersonal relationships is partly based on the relationships between interpersonal theories. It aims to help patients and caregivers build positive interpersonal relationships based on mutual respect and trust, assist patients in overcoming negative emotions, and break bonds of low self-esteem. By utilizing observational, empirical, and reflective approaches to structured and unstructured interactions, Peplau’s theory provides nurses with guidance in constructing strategic communication with patients. Anxiety plays a significant role in the interpersonal interaction process created by Peplau, and, if carefully addressed, can be the key to addressing a patient’s health issues [29]. Through the four stages of “recognition,” “determination,” “progression,” and “resolution,” dynamic communication can fill in the gaps in the patients’ knowledge about the disease, alleviate their concerns about upcoming anesthesia and surgery, and restore their confidence in their ability to overcome the illness. Nurses, especially nursing anesthetists assisting patients undergoing lung cancer surgery, play a critical role in the care of patients with lung cancer. They play a role in early task disclosure, patient care during surgery, escorting during wakefulness, post ward follow-up, and dynamic care that can readily address the patients’ first questions.

In summary, multimodal perioperative nursing based on Peplau’s interpersonal relationship theory may maintain a good nurse-patient relationship, dynamic communication, meet patient needs, reduce patient anxiety, and reduce disease uncertainty through comprehensive and personalized nursing between nurses and patients. A good psychological state of the patient will inevitably promote the physiological recovery of the body. Therefore, under this multimodal nursing plan, the postoperative rehabilitation quality indicators of patients in the PG patients also showed statistical significance. In addition to pain and physical comfort, physical comfort also demonstrated a good recovery trend. Previous studies have demonstrated that illness uncertainty is closely related to the patients’ quality of life [15, 30, 31]. These studies have found a negative correlation between illness uncertainty and quality of life, which can directly or indirectly affect the QoR of patients through perceived stress, depression, social support, and coping mechanisms. However, no significant difference was found in pain, length of PACU and hospital stay, and incidence of complications among patients. This could be attributed to the fact that the included patients were those with early-stage lung cancer, with relatively small surgical trauma and good rehabilitation effects. Additionally, the popularity of multimodal analgesia may be another reason, therefore, there was no statistically significant difference in pain scores between the two groups of patients.

This study had some limitations. Firstly, this is a retrospective study with a single center and small sample size. Due to non matching studies, there are many confounding factors, and the explanatory power of some observation indicators is insufficient. Secondly, a multimodal perioperative nursing observation based on Peplau’s interpersonal relationship theory was only carried out for a specific population of patients with surgical lung cancer. The population is limited, and the number of patients included in this study tends to be younger, which limits the generalizability and application of this nursing plan. Finally, implementing multimodal perioperative care based on Peplau’s interpersonal relationship theory may result in additional labor and time costs. Therefore, in the future, large sample, prospective, multicenter, randomized controlled studies are needed to confirm the effectiveness of this nursing model and evaluate its feasibility as a perioperative nursing plan.

## Conclusions

The multimodal perioperative nursing based on Peplau’s interpersonal relationship theory not only reduces the illness uncertainty of patients with lung cancer surgery and improves their QoR but also expands the application of this theory in clinical practice, guiding perioperative nursing of patients with lung cancer. However, due to the limitations of single center, small sample, and retrospective studies, further research is needed to verify the widespread clinical application of a multimodal perioperative nursing plan based on this theory.

## Data Availability

The authors confirm that the data supporting the findings of this study are available within the article.

## Abbreviations

NSCLC: Non-small cell lung cancer
QoR: Quality of recovery
PG: Peplau’s group
CG: Control group
AIDET: Acknowledge; introduce; duration; explicit; thank you
MUIS: Mishel uncertainty in illness scale
VAS: Visual analog scale
EA: Emergence agitation
SD: Standard deviation
TNM: Tumor Node Metastasis
PACU: Post-anesthesia care unit
